# Estimation of Fetal Foot Length and Femur-to-Foot Length Ratio in Indian Population for Estimating Gestational Age on Sonography During Second Trimester (17-25 Weeks)

**DOI:** 10.7759/cureus.84515

**Published:** 2025-05-20

**Authors:** Gautam Ravisankar, S Jaiganesh Sivalingam, BH Parameshwar Keerthi, Sundararajan Srinivasan

**Affiliations:** 1 Radiodiagnosis, Meenakshi Academy of Higher Education and Research, Kanchipuram, IND; 2 Radiology, Meenakshi Medical College Hospital and Research Institute, Kanchipuram, IND

**Keywords:** femur length, foot length, gestational age, ratio, ultrasonography

## Abstract

Background

Assessing accurate fetal gestational age is vital in ascertaining the accurate growth of the fetus. Even if there are multiple ways to evaluate the fetal gestational age using ultrasonography, foot length measurement of the fetus can be utilized as an alternate tool to ascertain the age of gestation accurately. The length of the fetal femur and foot was estimated and recorded, and their ratio was calculated in this study. This parameter helps in distinguishing dysplastic short limbs from those shortened due to constitutional factors or due to intrauterine fetal growth retardation. Ultrasonography was used in this study to assess the association between gestational age and fetal foot length. The aim of the study is to derive normogram correlating gestational age (in weeks) with fetal foot length and to derive the femur-to-foot length ratio in women with 17-25 weeks of gestation.

Materials and methods

In our study, 150 healthy women with singleton pregnancy and 17-25 weeks of gestation underwent routine ultrasound examination in the Department of Radio Diagnosis, Meenakshi Medical College Hospital and Research Institute. In addition to the routine parameters, foot length and femur-to-foot length ratio were estimated.

Results

A strong correlation (positive) was observed in this study with Pearson’s correlation coefficient of 0.996 seen between gestational age and foot length, and 0.930 between foot length and femur length, both with a statistically significant *p *value < 0.0001. The femur-to-foot length ratio ranged from 0.9 to 1 in all cases.

Conclusion

The study revealed a robust correlation, which was linear, between gestational age and foot length, thereby supporting the use of fetal foot length as an added biometric measure to estimate gestational age. Additionally, the study highlights that the femur-to-foot length ratio approximates 1, with a ratio below 0.92 serving as a reliable indicator for detecting most cases of dysplasia.

## Introduction

Evaluating normal fetal growth patterns and accurate gestational age (GA) estimation has long intrigued researchers and healthcare professionals worldwide. Precise GA estimation plays a pivotal role in managing pregnancy-related complications. Over the past century, various methods have been developed to measure fetal growth and its progression. Among these, the Carnegie staging system, first introduced by George L. Streeter, is particularly noteworthy. Subsequently, obstetric ultrasound (USG) emerged as the most widely used non-invasive modality for estimating fetal GA. Commonly used biometric parameters include head circumference (HC), biparietal diameter (BPD), abdominal circumference (AC), and femur length (FL). However, assessing GA estimation becomes challenging in fetuses with anomalies such as anencephaly, hydrocephalus, or short-limb dysplasia. BPD may yield inaccurate results in an unusually rounded (brachycephalic) or elongated (dolichocephalic) head. Variations in AC due to changes in liver size or subcutaneous fat in growth-restricted or macrosomic fetuses may affect precision. Achondroplasia may cause underestimation of FL, leading to incorrect GA assessment. Fetal foot growth follows a consistent, measurable pattern, making it a reliable GA marker [1]. Mercer et al. found foot length a credible parameter for GA estimation [2], and Platt et al. demonstrated its strong correlation with menstrual age. Furthermore, the femur-to-foot length ratio helps distinguish limb shortening due to skeletal dysplasia from that caused by constitutional factors or intrauterine growth restriction (IUGR) [3]. This study aimed to assess fetal foot length and calculate the fetal femur-to-foot length ratio, in addition to conventional parameters, to estimate GA in pregnancies between 17 and 25 weeks.

## Materials and methods

This retrospective observational study was conducted for a period of six months from November 2024 to April 2025 on 150 healthy women with singleton pregnancies between 17 and 25 weeks of gestation who underwent routine antenatal targeted imaging for fetal anomalies (TIFFA) scans at the Department of Radio-Diagnosis, Meenakshi Medical College Hospital and Research Institute, Kanchipuram. Ethical committee approval was obtained. Gestational age (GA) was estimated based on the last menstrual period (LMP). Participants with oligohydramnios, polyhydramnios, IUGR, or skeletal dysplasia were excluded. Ultrasound (USG) examinations were performed using a Samsung HS70 machine (Samsung Medison Co., Ltd., Seoul, South Korea) with a 4-18 MHz curvilinear transducer. Patients underwent TIFFA scanning by prior appointment. Each scan began with a preliminary fetal survey, including assessing fetal lie, placental position, amniotic fluid volume, and fetal heart rate. Biometric measurements were recorded. Biparietal diameter (BPD) and occipitofrontal diameter (OFD) were used to calculate the cephalic index and HC. Abdominal circumference (AC) was derived from the anteroposterior abdominal diameter (APD) and transverse abdominal diameter (TAD).

**Figure 1:**
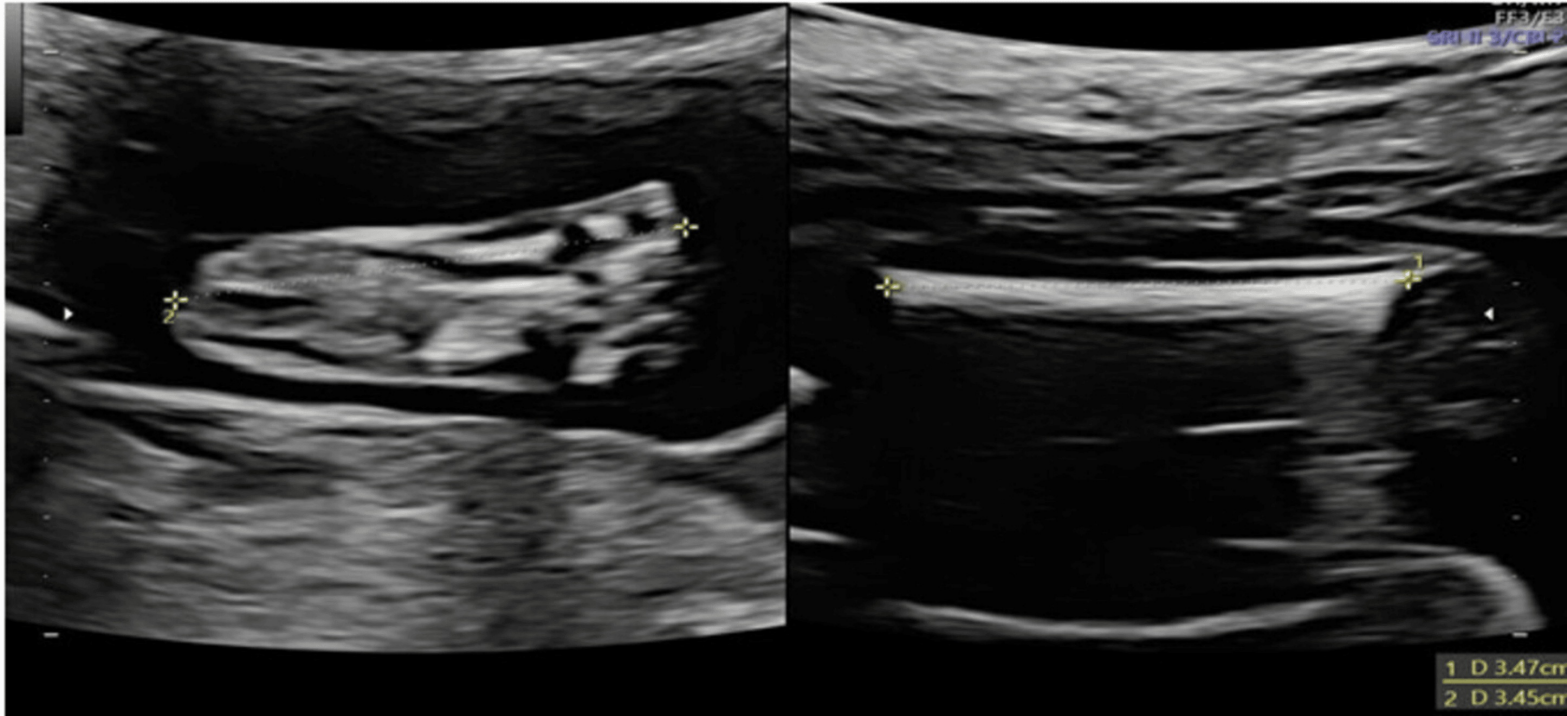
Ultrasonography (USG) image showing the foot (plantar view) and femur.

An image showing the full length of the ossified diaphysis was obtained to estimate FL, and its longest axis was measured. Calipers were placed at the tip of the ossified diaphysis, excluding the visible distal femoral epiphysis. Fetal foot length was measured from the skin edge at the heel to the tip of the longest toe (either the first or the second), as observed in the plantar or sagittal views. Figure 1 illustrates the appropriate method for measuring fetal FL and foot length.

Data were collected and analyzed using Statistical Package for the Social Sciences (SPSS) version 21.0 (IBM Corp., Armonk, NY, USA) and Microsoft Excel (Microsoft Corp., Redmond, WA, USA). Table 1 presents the collected measurements. The relationships between GA, foot length, and FL were analyzed using Pearson’s correlation and linear regression. Statistical significance was set at p<0.0001. The femur-to-foot length ratio was also calculated.

**Table 1:** Mean foot length and femur-to-foot length ratio at various gestational ages. SD: standard deviation

S.NO	GESTATIONAL AGE	NUMBER OF CASES	MEAN FOOT LENGTH (FTL)+ SD	MEAN FEMUR FOOT LENGTH RATIO
1	17 WEEKS	3	25.0 ± 1.7	0.9
2	18 WEEKS	7	28.8 ± 1.2	0.9
3	19 WEEKS	26	31.7 ± 1.6	1.0
4	20 WEEKS	39	34.7 ± 1.8	1.0
5	21 WEEKS	38	36.0 ± 2.3	1.0
6	22 WEEKS	18	39.3 ± 2.8	1.0
7	23 WEEKS	8	41.0 ± 2.7	0.9
8	24 WEEKS	6	44.0 ± 1.4	0.9
9	25 WEEKS	5	47.4 ± 1.1	0.9

## Results

**Figure 2:**
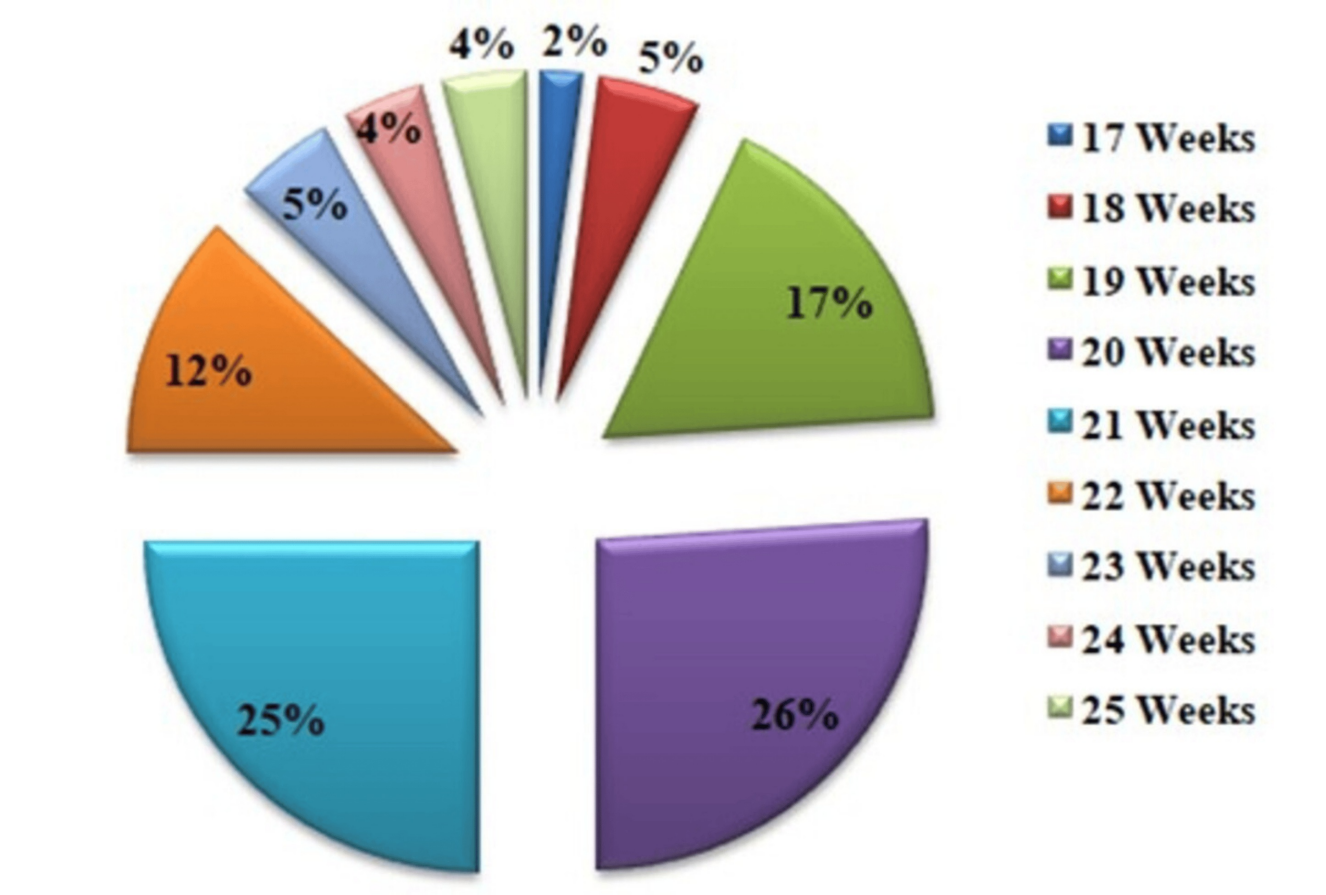
Pie chart showing the distribution of patients between 17-25 weeks of gestation.

The study included 150 healthy singleton pregnancies (primigravida or multigravida), with maternal age ranging from 19 to 35 years (mean: 27.3 years). Most scans were performed at 20 weeks (n=39; 26%), followed by 21 weeks (n=38; 25.3%). Only three patients underwent an early targeted scan at 17 weeks (n=3; 2%) (Figure 2).

**Figure 3:**
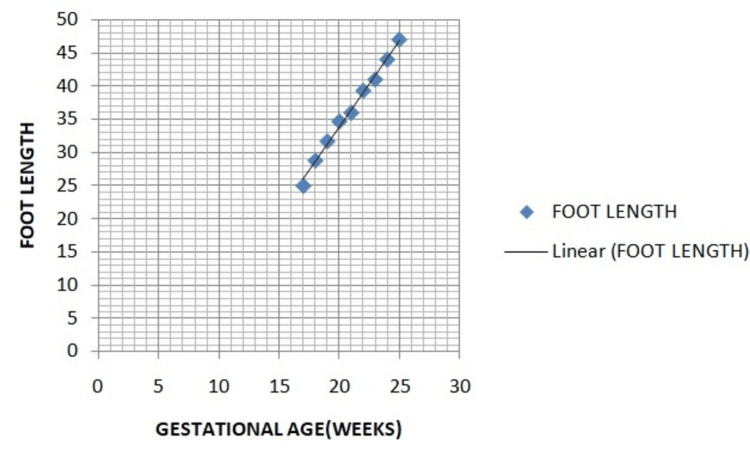
Regression plot showing the linear association between gestational age (GA) and mean foot length.

Simple linear regression formula calculated with T test simple linear regression formula revealed a strong positive correlation between foot length and GA (foot length in mm = 2.6133 x GA - 18.491), with a correlation coefficient of r = 0.996 (p<0.0001) (Figure 3).

**Figure 4:**
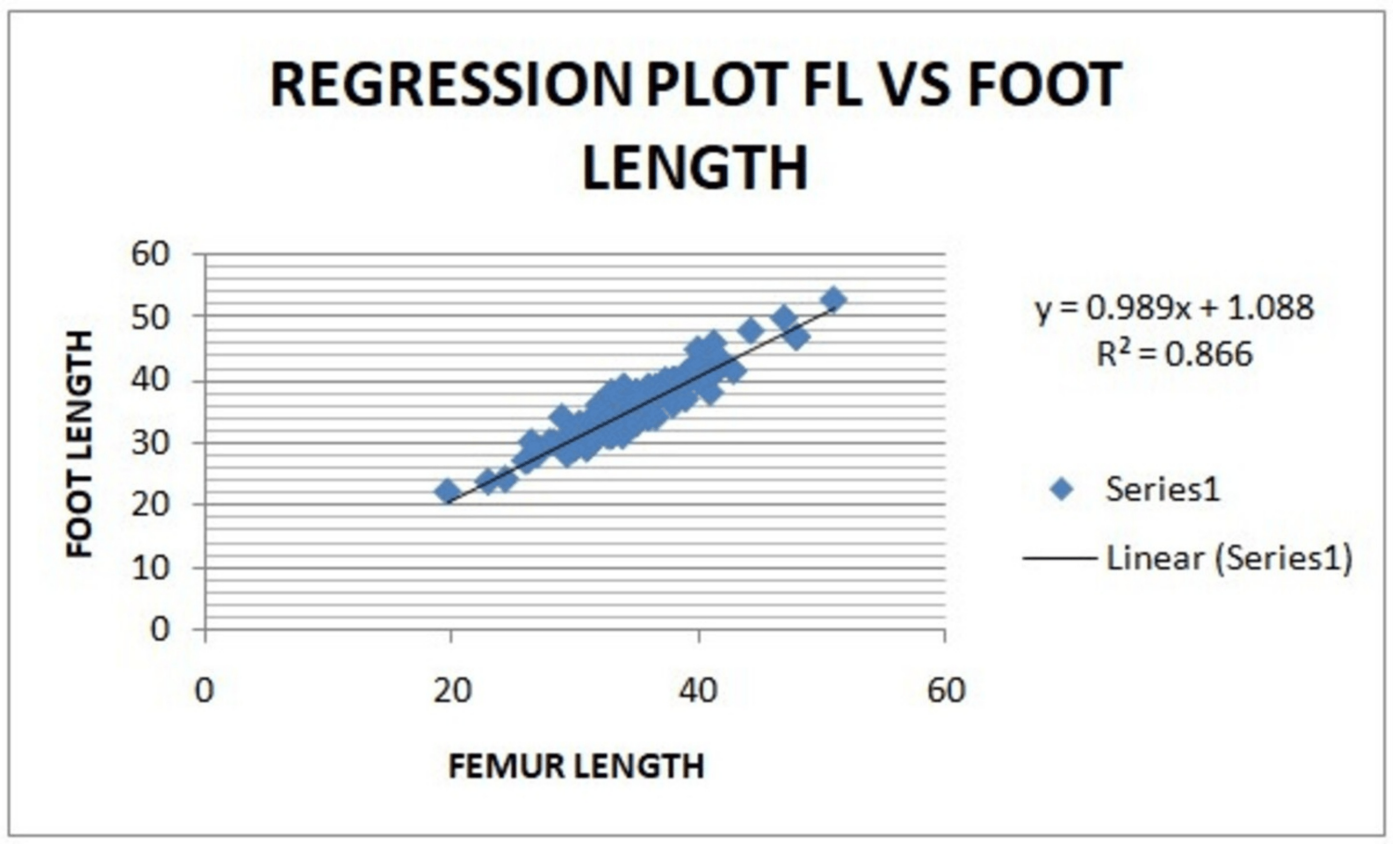
Regression plot of femur length versus foot length. FL: Femur length

On Pearson correlation coefficient, a strong correlation was observed between foot length and FL (foot length in mm = 0.9898 x FL + 1.0886) with r = 0.930 (p<0.0001) (Figure 4).

The associations between fetal FL, foot length, and GA are summarized in Table 2. The femur-to-foot length ratio remained relatively constant across GAs.

**Table 2:** Summary of fetal foot length, gestational age, and femur length relationship.

Y-AXIS	X-AXIS	Regression Formula	Correlation Coefficient	P-value
Foot measurement in length	Age of gestation	2.6133 x age of gestation (weeks) - 18.491	0.996	p<0.0001
	Femur measurement in length	0.9898 x age of gestation (weeks) + 1.0886	0.930	p<0.0001

## Discussion

Accurate estimation of GA is vital for effective obstetric management and optimal perinatal outcomes. Traditionally, GA is estimated using the LMP and Naegele’s rule. However, this method is frequently unreliable, particularly when women cannot recall their LMP accurately or have irregular menstrual cycles. Discrepancies in LMP-based dating can lead to inaccuracies, especially in cases involving multiple fetuses or amniotic fluid abnormalities such as oligo or polyhydramnios. In current clinical practice, USG has become the standard for estimating GA. It offers a reliable, non-invasive estimate and serves as a baseline for monitoring fetal growth throughout pregnancy, thereby ensuring high-quality antenatal and perinatal care [4].

Commonly used biometric parameters for estimating GA include the gestational sac and crown-rump length during the first trimester, and HC, BPD, AC, and FL in the second trimester. However, these parameters may be inaccurate in certain conditions such as macrocephaly, hydrocephalus, anencephaly, short-limb dwarfism, or IUGR. For instance, HC and BPD measurements may be affected by fetal head enlargement or abnormal positioning; FL measurements can also be imprecise in cases of short-limb dwarfism, and AC may not accurately reflect fetal size in fetal growth restriction (FGR) or macrosomia associated with gestational diabetes mellitus. These limitations necessitate the use of alternative markers for more accurate gestational dating, even in seemingly uncomplicated pregnancies [5].

Fetal foot length is a promising alternative. Based on our findings, foot length measurement is a simple, reproducible technique that can be easily integrated into routine obstetric USG examinations and yields reliable estimates of GA.

Shalev et al. demonstrated the accuracy of sonographic measurements of fetal foot length with promising results [6]. Our study further substantiates these findings, revealing a strong linear correlation between fetal foot length and GA, consistent with earlier reports, confirming its utility when other conventional parameters are inconclusive [6].

A recent study by Joshi et al. aligns with our conclusions [7]. Furthermore, Campbell et al. reported that the femur-to-foot length ratio remains approximately 1 throughout gestation (14-40 weeks) [8]. This ratio aids in distinguishing fetuses with skeletal dysplasia from those with constitutional smallness or symmetrical IUGR. Specifically, a ratio ≥0.9 is seen in constitutionally small fetuses or those with symmetrical IUGR. In contrast, a ratio <0.9 suggests skeletal dysplasia, characterized by limb shortening with relative preservation of the hand and foot lengths. Our study found similar results, with the normal fetal femur-to-foot length ratio between 0.9 and 1.1 [8]. Unlike the study by Joshi et al., fetuses with known foot length anomalies, such as short-limb dysplasia, were excluded from our study [7]. Johnson et al. demonstrated the utility of this ratio in identifying fetuses at increased risk for trisomy 21. However, our study focused exclusively on fetuses with femur lengths appropriate for GA, and cases of non-lethal or lethal skeletal dysplasia were not included. While Johnson et al. provided insights into abnormal foot length patterns, this study aimed to validate this ratio in structurally normal fetuses [9]. Our findings are consistent with those of Joshi et al. and Mital et al., who reported femur-to-foot length ratios of ≥0.9 in all cases studied [7,10].

Using scatter plots, Tikmani et al. reported a positive linear relationship between GA and foot length. Our results mirrored this correlation, demonstrating statistical significance (p<0.0001). However, unlike their broader inclusion criteria, our study focused specifically on normal intrauterine fetuses between 17 and 25 weeks of gestation - an important consideration, as certain countries permit termination for fetal anomalies up to 24 weeks [11].

## Conclusions

This study demonstrated a strong linear correlation between fetal foot length and GA and femur length. Accordingly, fetal foot length serves as a reliable alternative for estimating GA when standard methods are impractical, such as in cases of fetal hydrocephalus or anencephaly. Furthermore, a femur-to-foot length ratio ≥0.9 is typically considered normal in constitutionally small fetuses, provided mild IUGR is excluded.

## References

[1] Streeter GL (1920). Weight, sitting height, head size, foot length, and menstrual age of the human embryo. Contributions to Embryology.

[2] Mercer BM, Sklar S, Shariatmadar A, Gillieson MS, D'Alton ME (1987). Fetal foot length as a predictor of gestational age. Am J Obstet Gynecol.

[3] Platt LD, Medearis AL, DeVore GR, Horenstein JM, Carlson DE, Brar HS (1988). Fetal foot length: Relationship to menstrual age and fetal measurements in the second trimester. Obstet Gynecol.

[4] Hemraj S, Acharya DK, Abraham SM, Vinayaka US, Ravichandra G (2017). Fetal foot length and its sonographic correlation with gestational age. Donald Sch J Ultrasound Obstet Gynecol.

[5] Pandey VD, Singh V, Nigam GL, Usmani Y, Yadav Y (2015). Fetal foot length for assessment of gestational age: a comprehensive study in north India. Sch J App Med Sci.

[6] Shalev E, Weiner E, Zuckerman H, Megory E (1989). Reliability of sonographic measurement of the fetal foot. J Ultrasound Med.

[7] Joshi KS, Marahatta B, Karki S, Tamrakar S, Shrestha NC (2012). Fetal foot length and femur/ foot length ratio: significance in Nepalese context. Nep J Radiol.

[8] Campbell J, Henderson A, Campbell S (1988). The fetal femur/foot length ratio: a new parameter to assess dysplastic limb reduction. Obstet Gynecol.

[9] Johnson MP, Barr M, Treadwell MC (1993). Fetal leg and femur/foot length ratio: a marker for trisomy 21. Am J Obstet Gynecol.

[10] Mital M, Gupta P, Nanda V (2014). Fetal gestational age estimation by fetal foot length measurement and fetal femur to foot length ratio in Indian population - a prospective study. J Evol Med Dent Sci.

[11] Tikmani SS, Roujani S, Azam SI (2020). Relationship between foot length and gestational age in Pakistan. Glob Pediatr Health.

